# Expression profiles for macrophage alternative activation genes in AD and in mouse models of AD

**DOI:** 10.1186/1742-2094-3-27

**Published:** 2006-09-27

**Authors:** Carol A Colton, Ryan T Mott, Hayley Sharpe, Qing Xu, William E Van Nostrand, Michael P Vitek

**Affiliations:** 1 Duke University Medical Center, Division of Neurology, Durham, NC 27710, USA; 2 University of Bath, Department of Biology and Biochemistry, Clavertone Down, Bath, BA2 7AY, UK; 3 Department of Medicine, Stony Brook University, Stony Book NY 11794, USA

## Abstract

**Background:**

Microglia are associated with neuritic plaques in Alzheimer disease (AD) and serve as a primary component of the innate immune response in the brain. Neuritic plaques are fibrous deposits composed of the amyloid beta-peptide fragments (Abeta) of the amyloid precursor protein (APP). Numerous studies have shown that the immune cells in the vicinity of amyloid deposits in AD express mRNA and proteins for pro-inflammatory cytokines, leading to the hypothesis that microglia demonstrate classical (Th-1) immune activation in AD. Nonetheless, the complex role of microglial activation has yet to be fully explored since recent studies show that peripheral macrophages enter an "alternative" activation state.

**Methods:**

To study alternative activation of microglia, we used quantitative RT-PCR to identify genes associated with alternative activation in microglia, including arginase I (*AGI*), mannose receptor (*MRC1*), found in inflammatory zone 1 (*FIZZ1*), and chitinase 3-like 3 (*YM1*).

**Results:**

Our findings confirmed that treatment of microglia with anti-inflammatory cytokines such as IL-4 and IL-13 induces a gene profile typical of alternative activation similar to that previously observed in peripheral macrophages. We then used this gene expression profile to examine two mouse models of AD, the APPsw (Tg-2576) and Tg-SwDI, models for amyloid deposition and for cerebral amyloid angiopathy (CAA) respectively. *AGI, MRC1 *and *YM1 *mRNA levels were significantly increased in the Tg-2576 mouse brains compared to age-matched controls while *TNFα *and *NOS2 *mRNA levels, genes commonly associated with classical activation, increased or did not change, respectively. Only *TNFα *mRNA increased in the Tg-SwDI mouse brain. Alternative activation genes were also identified in brain samples from individuals with AD and were compared to age-matched control individuals. In AD brain, mRNAs for *TNFα*, *AGI, MRC1 *and the chitinase-3 like 1 and 2 genes (*CHI3L1; CHI3L2*) were significantly increased while *NOS2 *and *IL-1β *mRNAs were unchanged.

**Conclusion:**

Immune cells within the brain display gene profiles that suggest heterogeneous, functional phenotypes that range from a pro-inflammatory, classical activation state to an alternative activation state involved in repair and extracellular matrix remodeling. Our data suggest that innate immune cells in AD may exhibit a hybrid activation state that includes characteristics of classical and alternative activation.

## Background

As part of the innate immune system, macrophages rapidly respond to a large variety of pathological molecular pattern stimuli (PAMPS) such as bacterial coat and viral proteins [1,2]. The programmed response to acute stimuli includes the induction of a specific gene profile and the subsequent production of multiple cytoactive factors such as TNFα, NO and IL-1 that protect against tissue invaders. In peripheral macrophages, this first phase of an innate immune response has been described as classical immune activation [1-3]. "Classical activation" is also characterized by the involvement of Th-1 cytokines such as interferon-γ (IFN-γ), a "master" cytokine that orchestrates the coordinated induction and production of the "killing" phase [4-6]. However, the gene profile of macrophages can change, shutting down the production of pro-inflammatory cytokines and increasing the production of factors that participate in tissue repair and wound healing [6]. Anti-inflammatory, Th-2 cytokines such as IL-10 and TGFβ are associated with broad ranging and potent inhibition of pro-inflammatory activity while other Th-2 cytokines such as IL-4 and IL-13 serve to antagonize IFN-γ, are anti-parasitic, mediate allergic responses and induce tissue matrix reconstruction [6,7]. IL-4 and IL-13 -mediated gene induction has been specifically termed alternative activation and includes genes that produce arginase I (*AG1*), mannose receptors (*MRC1*) and genes associated with tissue remodeling such as Found in Inflammatory Zone 1 (*FIZZ1*) and chitinase 3-like 3 (*YM1*) [8-11].

The induction of the genes characteristic of alternative activation during an immune response provides an anti-inflammatory balance to an acute, pro-inflammatory response. Consequently, alternatively activated macrophages are viewed as immunosuppressive and involved in tissue repair and extracellular matrix remodeling [5,6]. However, alternatively activated macrophages may also contribute to disease processes in a complex way. For example, alternatively activated alveolar macrophages contribute to the fibrotic lesion in idiopathic pulmonary fibrosis [11] and in the liver fibrosis associated with *Schistosoma mansoni *[12].

Although classical and alternative activation are commonly viewed as the two opposing ends of macrophage activation, additional activation states may also exist [5,6]. For example, Anderson and Mosser [13] have described a third class of macrophages, called Type II macrophages. This state requires a specific two step activation pattern that involves ligation of Fcγ receptors and signaling through Toll receptors, CD40 or CD44 [5,13,14]. The end result is decreased *IL-12 *expression concomitant with increased *IL-10 *mRNA. As a consequence, the gene profile of Type II macrophages is a mixture of pro-inflammatory and anti-inflammatory genes such as *IL-1, TNF-a, IL-6, IL-10 *and *IL-4 *[15]. Arginase, however, is not induced. A "deactivation" state of macrophages that is similar to Type II macrophages has also been described by Gordon [6].

Alzheimer's disease (AD) is characterized pathologically by extracellular fibrillar deposits in the parenchyma of the brain which are composed of the β-amyloid (Aβ) peptide 1–40 and 1–42 fragments of the amyloid precursor protein (APP) [16-18]. It is generally believed that soluble APP and various forms of Aβ peptides, either alone or in conjunction with other immune factors, serve as activating signals for an innate immune response in the brain [19]. Using immunocytochemistry, Griffin et al [20] have demonstrated the presence of IL-1β in microglia and astrocytes surrounding the amyloid deposits. Other investigators have confirmed these findings and have also shown that IL-6, TNFα and MHC expression is increased in AD [21-25]. As a result, AD has been associated with classical immune activation and the production of an acute Th-1 immune response. However, AD is a chronic neurodegenerative disease in which the inflammatory process has not been thoroughly charted over time and with disease progression. It is highly likely that brain macrophages may change their activation state as a function of the disease and time. To assess this possibility, we have determined if genes associated with alternative activation are expressed in cortical samples from individuals with AD compared to cognitively normal aged matched control individuals. Additionally, we investigated the alternative activation-related gene expression profiles in mouse models of AD and of cerebral amyloid angiopathy. Our data demonstrate that genes typical of alternative activation are clearly expressed in AD and in a mouse model of amyloid deposition. However, it is likely that the macrophage activation state in AD represents a novel hybrid state between classical and alternative activation.

## Materials and methods

### Cell cultures and treatment conditions

BV2 microglia were used as a model of CNS murine microglia and have been extensively characterized [26]. For some experiments, primary murine microglia were also used and were prepared from mixed glial cultures from 2 day old postnatal pups in a standard fashion [27]. Cells from both cultures were maintained in 75 cm^2 ^flasks at 37°C in a 5% CO_2 _humidified atmosphere in Dulbecco's Modified Eagle Medium (DMEM) with high glucose (4.5 g/L D-glucose, L-glutamine, pyridoxine HCl, and 110 mg/L sodium pyruvate; Invitrogen, Carlsbad, CA, USA) containing 10% fetal bovine serum, 100 U/ml penicillin, and 100 μg/ml streptomycin. For each experiment, cells were plated into 24 well dishes, after which the media was changed to the treatment media consisting of serum free DMEM with low glucose (1.0 g/L D-glucose, L-glutamine, pyridoxine HCl, and 110 mg/L sodium pyruvate; Invitrogen). Cells were then allowed to adapt to the serum-free media for 24 hours before starting the experiments. The stimulants used for the cell culture experiments included recombinant mouse IFN-γ (100 U/ml, BioSource International Inc., Camarillo, CA, USA) and recombinant mouse IL-4 or IL-13 (20 ng/ml, BioSource International). Treatments were carried out in fresh serum-free media for 24 hours at 37°C in a 5% CO_2 _humidified atmosphere, after which the assays were performed. All experiments were repeated a minimum of three times.

### Transgenic mice

Transgenic mice (Tg-2576) containing the Swedish (K670N/M671L) APP double mutation were generously provided by Dr. Karen Hsiao-Ashe. Tg-SwDI mice containing the Swedish, and the CAA-associated Dutch (E22Q) and Iowa (D23N) APP mutations, were generated as described [28]. Tg-2576 mice, together with wild-type controls were maintained until 70 weeks of age. Tg-SwDI mice and their wild-type controls were maintained until 60 weeks of age and were of mixed gender. The mice were sacrificed and their brains were removed, snap-frozen in liquid nitrogen, and stored at -80°C. To isolate total RNA, cortical forebrain samples (approximately 100 mg) were homogenized in 1 ml of Trizol and extracted with 200 μl of chloroform. The aqueous phase was separated by centrifugation (12,000 × g for 15 minutes at 4°C), mixed with an equal volume of 70% ethanol, and purified using the RNeasy mini-kit (QIAGEN Inc., Valencia, CA, USA). Synthesis of cDNA from the total RNA samples was performed using the High Capacity cDNA Archive Kit (Applied Biosystems, Foster City, CA, USA).

### Human brain tissue

Rapid autopsy human brain samples were obtained from Kathleen Price Bryan Brain Bank under Duke IRB approval. For this study, frozen frontal lobe (cortical) brain tissue samples were prepared from autopsy of individuals with pathologically confirmed AD and age-matched, cognitively normal control patients. Characteristics of the sample population are shown in Table 1. Average age and average post-mortem interval (PMI) were not significantly different between normal and AD populations. All normal individuals were diagnosed as Braak and Braak stage 1 while AD individuals were diagnosed as Braak and Braak stage IV or V. For each specimen, cortical gray matter was carefully dissected so as to minimize inclusion of white matter and subarachnoid blood vessels. Total RNA was isolated and converted to cDNA as described above.

**Table 1:** AD and normal control brain – characteristics.

	**Age (yrs)**	**Gender**	**PMI (hrs)**	**Braak & Braak score**	*APOE 4 *gene*
**Normal control**	78.3 ± 1.7 (29)	12 Male 17 Female	9.1 ± 1.6	Stage 1	5(*APOE 3/4*)
**AD**	77.8 ± 1.0 (47)	23 Male 24 Female	6.8 ± 1.4	Stage 4–5	13 (*APOE3/4*) 14(*APOE4/4*)

*Number of individuals expressing an APOE4 gene

### Real-time PCR

Real-time PCR was performed using the TaqMan Gene Expression Assay Kit (Applied Biosystems) according to the manufacturer's instructions. Briefly, cDNA samples (100 ng, based on the original RNA concentrations) were brought to a total volume of 22.5 μl using RNase-free water and mixed with 25 μl of 2X TaqMan Universal Master Mix (without AmpErase uracil-N-glycosylase) and 2.5 μl of the respective 20X TaqMan Gene Expression Assay. Target amplification was performed in 96-well plates using a real-time sequence detection system instrument (ABI PRISM 9700HT, Applied Biosystems). The PCR thermal cycling conditions included an initial 10 minute hold at 95°C to activate the AmpliTaq Gold DNA polymerase, followed by 40 cycles of denaturation (15 seconds at 95°C) and annealing/primer extension (1 minute at 60°C). The data from the real-time PCR experiments were analyzed using the 2^-ΔΔCt ^method, which allows for the calculation of relative changes in gene expression [29]. For this method, the threshold cycle number (Ct) is normalized using a housekeeping gene (18s rRNA), calibrated to the control samples, and the result used as the exponent with a base of 2 to determine the fold change in gene expression. The treatment conditions used in this study did not alter the expression of 18s rRNA, thus validating its use as a normalizing factor. Untreated BV2 cells, littermate wild type mouse brains or non-AD, age matched human brain served as the comparator where appropriate. Primers for these experiments were purchased from Applied Biosystems Foster City, CA, USA. Table 2 provides the Applied Biosystems ID number for each gene and information on exact primer sequences is provided through the Applied Biosystems Web site [30].

**Table 2:** Primer ID list. All primers were purchased from Applied Biosystems, Foster City, CA

**Mouse Primer/Probes**		
**Gene**	**origin**	**Applied Systems Batch ID**

18s (Eukaryotic 18s rRNA)	ms	Hs99999901_s1
Arg1 (arginase 1, liver)	ms	Mm00475988_m1
Arg2 (arginase2, type II)	ms	Mm00477592_m1
Chi3l3(chitinase 3-like 3, Ym1)	ms	Mm00657889_mH
IL1b (Interleukin 1 beta)	ms	Mm00434228_m1
Mrc1 (mannose receptor, C type 1)	ms	Mm00485148_m1
Nos2 (Nitric oxide synthase 2, inducible, macrophage)	ms	Mm00440485_m1
Ptprc (protein tyrosine phosphatase, receptor type, C, CD45)	ms	Mm00448463-m1
Retnla (resistin like alpha, FIZZ1)	ms	Mm00445109_m1
Slc7a2 (solute carrier family 7 (cationic amino acid transporter, y+system), member 2, Cat2)	ms	Mm00432032_m1
Slc7a3 (solute carrier family 7 (cationic amino acid transporter, y+system), member 3, Cat3)	ms	Mm00500256_m1
Tnf (tumor necrosis factor)	ms	Mm00443258_m1

**Human Primer/Probes**		

**Name**	**origin**	**Batch ID**

hARG1 (arginase, liver)	hu	Hs00163660_m1
hARG2 (arginase, type II)	hu	Hs00265750_m1
hCHI3L1 (chitinase 3-like 1 (cartilage glycoprotein-39))	hu	Hs00609691_m1
hCHI3L2 (chitinase 3-like 2)	hu	Hs00187790_m1
hIL1B (interleukin 1, beta)	hu	Hs00174097_m1
hMRC1 (mannose receptor, C type 1)	hu	Hs00267207_m1
hNOS2A (nitric oxide synthase 2A (inducible, hepatocytes))	hu	Hs00167248_m1
hPTPRC (protein tyrosine phosphatase, receptor type, C, hCD45)	hu	Hs00174541_m1
hSLC7A2 (solute carrier family 7 (cationic amino acid transporter, y+ system), member 2, hCAT2)	hu	Hs00161809_m1
hSLC7A3 (solute carrier family 7 (cationic amino acid transporter, y+ system), member 3, hCAT3)	hu	Hs00364157_m1
hTNF (tumor necrosis factor (TNF superfamily, member 2))	hu	Hs00174128_m1

### Statistical analyses

The data were analyzed using GraphPad Software (PRIZM) (San Diego, CA). One-way analysis of variance (ANOVA) was used to compare the means of the cell culture treatment groups. The data for the mouse (mutant versus wild-type) and human brain (AD versus control) samples were analyzed using either unpaired Student's t-test or the Wilcoxon Test, depending on whether the populations had equal variances as determined using the F-test.

## Results

**Figure 1:**
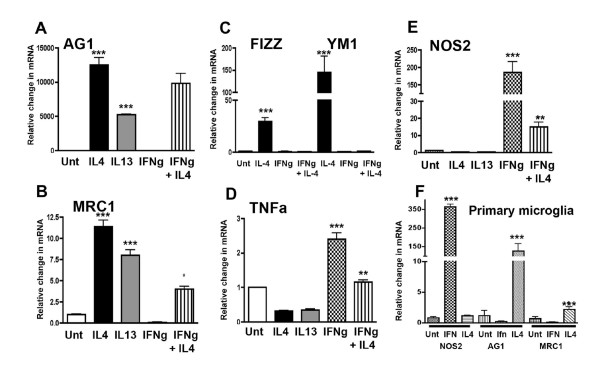
**Alternative activation genes are induced by treatment of microglia with IL4 or IL-13**. BV2 cells were treated with IL-4 or IL-13 for 24 hrs and the mRNA expression levels for *AG1 *(Panel A); *MRC1 *(Panel B); *FIZZ *and *YM1 *(Panel C) were determined using quantitative RT-PCR. mRNA levels for each of these genes significantly increased compared to untreated alone. IL-4 or IL-13 treatment failed to induce *TNFα *(Panel D) or *NOS2 *(Panel E) *mRNA *expression. To determine if the alternative activation genes were induced by classical activation agents, cells were treated with IFNγ (panels A-C). In this case, no induction was observed with IFNγ treatment and, with the exception of *AG1 *(A), treatment of BV2 cells with the combination of IFNγ and IL-4 reduced mRNA expression of each gene studied; Panel F- Primary microglia obtained from neonatal mouse cortex also demonstrated increased mRNA expression for alternative activation genes (*AG1; MRC1*) on stimulation with IL-4. *NOS2 *was not increased by IL-4 treatment but was increased with IFNγ treatment. * = p < 0.001 compared to IL-4 treated alone; ** = p < 0.001 compared to IFNγ treated alone; *** = p < 0.001 compared to untreated alone.

Alternative activation gene expression profiles have been described for tissue macrophages found in the periphery but have not been identified for brain macrophages, the microglia. Since induction of alternative activation has been linked to specific Th2 cytokines and in particular, to IL-4 and IL-13, we treated BV2 microglia with IL-4 (20 ng/ml) or IL-13 (20 ng/ml). We then used quantitative RT-PCR measurements to confirm the presence of specific genes known to be characteristic of alternative activation in IL-4/IL-13 treated cells compared to classically activated cells (treated with IFNγ) or untreated cells. As shown in Fig 1, BV2 cells demonstrate increased gene expression for *AG1, MRC1, FIZZ *and *YM1 *compared to untreated cells on induction by IL-4 or IL-13. In contrast, IFNγ treatment did not induce any of the alternatively activated genes but did induce *TNFα *and *NOS2 *mRNA, two well-described markers of classical activation. Co-treatment of IFNγ with IL-4 reduced the expression of *MRC1, FIZZ *and *YM1 *but not *AG1*, indicating that IFNγ can generally oppose IL-4 action. A similar effect was observed for *TNFα *and *NOS2 *mRNA where co-treatment with IL-4 plus IFNγ opposed, in this case, IFNγ-mediated induction. Primary microglia were also treated with either IL-4 or IFNγ to confirm the findings in BV2 microglia. As shown in Fig 1F, IFNγ induced an increased expression of *NOS2 *mRNA but did not affect either *AG1 *or *MRC1 *expression. In contrast, IL-4 treatment increased *AG1 *and *MRC1 *mRNA, suggesting that both BV2 microglia and primary microglia can demonstrate an alternative activation gene profile.

### Alternative activation gene profiles in mouse models of AD

**Figure 2:**
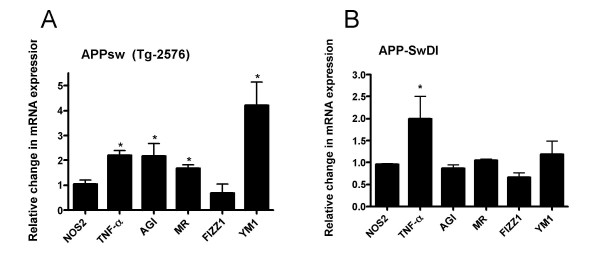
**Alternative activation genes in mouse models of amyloid pathology**. The transcripts of activation-related genes were measured in cortical extracts from Tg-2576 (AD model) and Tg-SwDI mice (CAA model) using quantitative RT-PCR. The data are presented as the average (± SEM) fraction of control levels where the appropriate aged-matched wild type littermate mice served as the comparator control. **A.Tg-2576 mice**- mRNA for *TNFα *(p < 0.01), *AGI *(p = 0.05), *MRC1 *(p < 0.01), and *YM1 *(p < 0.02) were significantly increased in the Tg-2576 mice brain **B. Tg-SwDI mice**- Only *TNFα *mRNA levels were significantly increased (p < 0.05) in Tg-SwDI mouse brain.

Since alternative activation could be demonstrated *in vitro *using the expression of specific genes, we determined if mouse models of amyloid deposition similar to AD exhibited an alternative activation gene profile. Two different transgenic mouse models were used, the APPsw Tg-2576 mouse containing the Swedish mutation [31,32] and the Tg-SwDI mouse model of cerebral amyloid angiopathy (CAA) [28,33]. Differences in the gene expression profiles between the mouse models were observed. In the Tg-2576 mouse (Fig 2A), among the alternative activation genes, *AG1, MRC1*, and *YM1 *demonstrated significant increases in expression, while *FIZZ1 *was expressed at wild-type levels. For genes commonly associated with classical activation, we found that *NOS2 *mRNA was expressed at wild-type levels while *TNFα *mRNA expression was slightly, but significantly elevated (Fig. 2A). Cortical extracts from Tg-SwDI mice were also examined. Pathologically, these mice have predominantly cerebrovascular amyloid and have high levels of immune reactive microglia localized to the cerebral blood vessels [33,34]. In comparing the six genes in the Tg-SwDI CAA mouse model, we observed a significant increase only in *TNFα *mRNA (Fig. 2B). The remaining genes failed to show a statistically significant difference between the mutant and wild-type animals.

### Brains from AD patients show increased gene expression of alternative activation markers

**Figure 3:**
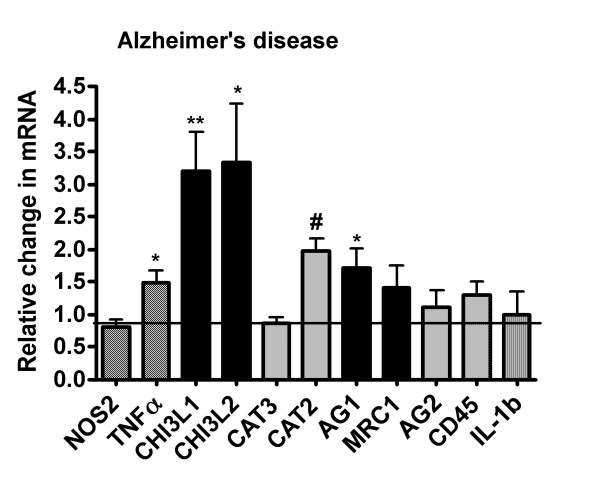
**Alternative activation genes in Alzheimer's disease**. mRNA expression was determined for *NOS2*, *TNFα, IL-1β*, *AGI, MRC1, CHI3L1, CHI3L2, AG2, CD45, CAT 2 *and *CAT3 *in frontal lobe cortical extracts from AD patients and age-matched, cognitively normal controls. The real-time PCR results are expressed as the average (± SEM) fraction of control where age-matched, cognitively normal brain served as the comparator control. The data show significant elevations in mRNA expression levels for *AG1 *(* = p < 0.04), *CHI3L1 *(** = p < 0.006), and *CHI3L2 *(* = p < 0.04). *NOS2 *and *IL-1β *mRNA did not change but *TNFα *mRNA increased significantly (* = p < 0.04) in AD. *CAT2 *mRNA, which encodes the inducible arginine transporter, also significantly increased (# = p < 0.02) in AD compared to control. *AG2, MRC1, CD45 *and *CAT*3 mRNA expression levels were equivalent between the AD and control brains.

To translate the data derived from cell culture and mouse models of AD, we measured expression levels of genes associated with classical and alternative activation in frontal lobe cortical extracts from AD patients and cognitively normal, age-matched controls individuals. As previously shown in Table 1, there were no significant differences between age or post-mortem interval (PMI) between groups. Messenger RNA expression levels were used rather than protein levels for detection of gene induction because RNA is typically less dependent on PMI than protein stability [35]. In addition, partial degradation of RNAs that may occur in post-mortem tissue does not obscure the reliable detection of specific mRNA transcripts using RT-PCR [35]. For the genes identified in classical activation, we found that *NOS2 *mRNA expression and *IL-1β *mRNA expression were not significantly different between control and AD, while *TNFα *mRNA was significantly increased in the AD group (Fig. 3). Among the alternative activation genes, we found that *AG1 *mRNA was increased approximately 2 fold in AD while *MRC1 *mRNA increased slightly but did not attain statistical significance due to a large variation in values. We were unable to detect expression of human *FIZZ1 *in any of the AD or control brain tissue samples. The remaining alternative activation gene, *YM1 *(also known as chitinase 3-like 3), has no direct human homologue. Thus, we selected two human genes closely related to *YM1*, that is; chitinase 3-like 1 (*CHI3L1*) and chitinase 3-like 2 (*CHI3L2*) [36]. We found that both *CHI3L1 *and *CHI3L2 *mRNAs were expressed at approximately 3 fold higher in AD brain compared to age matched controls. As an additional control, we also measured the expression of arginase 2 (*AG2*) mRNA, which encodes a mitochondrial isoform of arginase that is expressed in macrophages, neurons and astrocytes, but is not as commonly associated with immunological regulation as is *AGI *[37]. *AG2 *was expressed at equivalent levels between the AD and control samples, suggesting that the increase in *AG1 *mRNA was unlikely to be non-specific. Microglial and macrophage cell number were compared between control and AD tissue by measuring the expression of *CD45*, which is expressed on both resting and activated microglia [38,39]. In contrast to rodent brain [39], CD45 expression level is independent of activation state in human microglia [40]. Our data show that *CD45 *mRNA was expressed at equivalent levels between the AD and control groups and suggests that the numbers of microglia are grossly the same. A similar result was found for *CAT3*, a neuronal arginine transporter while cationic amino acid transporter 2 (*CAT2*), an immune inducible arginine transporter found in microglia, astrocytes and neurons [41], was also significantly increased in AD.

## Discussion

The regulation of gene transcription in macrophages during immune activation is dependent on multiple factors including the type of phagocytic ligands, the types of non-phagocytic adhesion interactions, the extracellular matrix and the cytokine environment [6,42]. Multiple, distinct populations of macrophages have been identified and include classically activated macrophages that are pro-inflammatory and associated with the "killing" phase of the innate immune response and alternatively activated macrophages that are primarily associated with wound healing and tissue repair. Type II macrophages that appear to be a hybrid activation state share some characteristics of each [5,6]. Peripheral macrophages cycle between these activation states, and dysregulation of this cycling underlies various forms of chronic disease [43].

To determine if innate immune cells of the brain exhibit alternative activation, we have used quantitative RT-PCR to identify a specific gene profile. This profile was created by stimulating BV2 and primary murine microglia with anti-inflammatory cytokines used to induce an alternative activation state in peripheral macrophages. Similar to peripheral macrophages, microglia treated with IL-4 or IL-13 significantly increased mRNA expression levels for *AG1*, *MRC1, FIZZ *and *YM1*, alternative activation genes. In contrast, genes for classical activation, *NOS2 *and *TNFα *, were not increased by either IL-4 or IL-13 treatment. Thus, our data confirm previously published studies on peripheral murine macrophages and provide a tool to assess the presence of alternative activation gene induction in the brain.

Using this gene expression pattern, we then probed cortical tissue from two transgenic mouse models of AD and from individuals with AD for evidence of alternative activation genes. These results are compared in Table 3. Essentially, cortical tissue from the Tg-2576 mouse and individuals with AD demonstrate a mixed profile of alternative activation and classical activation genes, particularly TNFα. The Tg-SwDI mouse that represents a cerebrovasuclar amyloid model, however, primarily demonstrates classical activation.

**Table 3:** Comparison of alternative activation gene profile.

	**Tg-2756**	**Tg-SwDI**	**AD**
***TNFα***	Increased	Increased	Increased
***NOS2***	No Change	No Change	No Change
***AG1***	Increased	No Change	Increased
***CHI3L1***	-	-	Increased
***CHI3L2***	-	-	Increased
YM1*	Increased	No Change	-
FIZZ1**	No Change	No Change	-
***MRC1***	Increased	No Change	No Change

* Rodent only- shares homology with CHI3-family

** Rodent only-shares homology with Resistin family

The presence of alternative activation genes in AD necessitates a more complex view of inflammation in neurodegenerative disease. Numerous studies have shown that the immune cells in the vicinity of amyloid deposits in AD express mRNA and proteins for pro-inflammatory cytokines, leading to the hypothesis that AD is primarily associated with classical (Th-1) immune activation [20,44-47]. Multiplex ribonuclease protection assays and gene micro-array studies have only partially confirmed this hypothesis [46,48,49]. For example, Blalock et al [48] have examined gene profiles found in AD using gene arrays on brain samples from 22 AD subjects. The primary pro-inflammatory genes represented were MHC class II and IFNγ, although IL-18 mRNA expression was elevated as well as genes for cytokine receptors, particularly IL-6R and IL-10R. A second array analysis by Xu et al [49] confirmed the increased in MHC class II but did not find changes in other pro-inflammatory genes. Coangelo et al [25], however, found a 3-fold increase in IL-1β expression using a DNA microarray analysis based on pooled AD brain samples compared to pooled control samples. The discrepancies and variability in gene expression patterns between the published studies are puzzling, especially in view of the fact that acute exposure of microglia to Aβ peptides initiates a classical activation pattern of gene expression [23,50-52]. There are many possible reasons for the differences between the *in vitro *and *in vivo *data. Hoozemanns et al[53] have suggested that the sequence and timing of pathological events in AD is critical. We further suggest that microglia may exhibit specific stages of response during a chronic neuroinflammatory disease such as AD. These stages in microglia in AD may be similar to those stages observed in chronic inflammatory diseases of the lung or liver, including the induction of alternative activation.

A shift to alternative activation in brain innate immune cells is supported by the analysis of mRNA expression for classical and alternative genes in the Tg-2576 mouse model of AD, a well-studied animal model for CNS parenchymal amyloid deposition [32]. *AGI, MR*C *1 *and *YM1 *mRNA levels were significantly increased in the Tg-2576 mouse brains compared to age-matched controls while *TNFα *and *NOS2*, genes commonly associated with classical activation, increased and did not change, respectively. The increased *TNFα *mRNA suggests a mixed activation state reminiscent of Type II macrophage activation [5]. However, both classical and Type II activated peripheral macrophages exhibit increased *NOS2 *mRNA, not decreased NOS2, and no induction of *AG1*. Thus, since *NOS2 *mRNA induction is not observed in peripheral macrophages that exhibit alternative activation, while *AG1 *expression is increased [5,54,55]., the preponderance of the data suggest that alternative activation is a dominant feature of the innate immune response in the APP Tg-2576 mouse. However, we cannot rule out that activation state in the APP Tg-2576 mouse is a novel, hybrid state.

In contrast, the Tg-SwDI mouse model, which represents a localized cerebrovascular amyloid angiopathy [33,34]., did not demonstrate the same increase in alternative activation markers. These differences may be due to the predominant cerebrovascular microglial proinflammatory phenotype that is observed in Tg-SwDI mice brains or in humans who express either the Iowa or Dutch mutation [33,56]. An increase in *TNFα *mRNA observed in the Tg-SwDI mice brains is consistent with this hypothesis.

Both classical and alternative activation markers were also observed in brains from AD patients and resemble the activation pattern found in Tg-2576 mice. In AD brain, mRNAs for *TNFα, AGI, CHI3L1 *and *CHI3L2 *were significantly increased in cortical samples compared to age-matched control brains while no significant difference was observed for *MRC1 *mRNA. The strong presence of the alternative activation genes in cortical tissue samples in AD brain implies that cells, such as microglia or astrocytes, have undergone a shift in functional profile. This finding does not negate immunocytochemical studies that demonstrate discretely localized, pro-inflammatory cytokine expression such as IL-1β in plaque associated microglia or astrocytes. However, the presence of alternative activation markers in plaque associated cells has not been determined. Thus, it is not clear if microglia within the vicinity of plaques show a complex activation state or if some cells express pro-inflammatory genes while others express alternative activation genes in a mosaic-like pattern.

The induction signal(s) for alternative activation in the amyloid mouse models and in AD remains unclear. Although IL-4 and IL-13 have been most closely linked to alternative activation [6], other Th-2 cytokines such as IL-10 and TGFβ down-regulate inflammation and are involved in repair and matrix remodeling [6,57-59]. Of these, only TGFβ has been firmly observed in AD brain [60,61]. while both TGFβ and IL-10 immunoreactivity have been detected in brains of Tg-2576 mouse [45]. The effects of anti-inflammatory cytokines in AD are largely unknown. Recently, however, Koenigsknecht-Talboo and Landreth [62] have shown that IL-4, IL-13, TGFβ or IL-10 enhance uptake of fibrillar Aβ peptides. Interestingly, no effect on Aβ uptake is observed with the anti-inflammatory cytokines alone, but instead, they serve to reduce the suppression of Aβ phagocytosis initiated by pro-inflammatory cytokines. These findings underscore the complexity of the brain's cytokine environment and its role in modifying microglial responses to Aβ peptides. Aβ, itself, may also influence the gene switch from classical towards alternative activation in microglia. Fibrillar Aβ interacts with numerous microglial membrane receptors including scavenger receptors A and B; CD40; an α6/β 1integrin/CD36/CD47 complex and complement receptors [63-67]. Crosslinking of these or other receptors has been associated with macrophage "down-regulation" through multiple mechanisms [68,69]. For example, mannose receptor signaling initiates an anti-inflammatory program within macrophages [68]. MRC-1 (CD206) is a transmembrane glycoprotein that mediates Ca^2+ ^dependent endocytosis and phagocytosis of mannosylated ligands [58] whose role in AD is currently unknown.

The induction of alternative activation genes is commonly considered to be a harbinger of repair and extracellular matrix re-organization that may begin during or after the first stages of an acute innate immune response [6,70,71]. Although the exact functions of many of the protein products of these genes are not clear, some alternative activation genes such as *AG1 *have been well studied in peripheral macrophages. Both isoforms of arginase utilize arginine as a substrate for biosynthetic pathways that produce polyamines and proline [72]. Polyamines such as spermine are well known to alter cell proliferation but have wide-ranging physiological effects such as regulation of NMDA channel function, membrane potentials and gene transcription [73,74]. Proline is an important component of collagens and is involved in repair of the extracellular matrix. The maintenance of high *AG1 *expression, as observed in our studies, is likely to direct arginine utilization toward the production of proline or polyamines and away from the production of nitric oxide. The enzymatic activities of both inducible NOS and arginase are solely dependent on intracellular arginine and these enzymes compete for arginine [72]. The low expression of *NOS2 *mRNA coupled with the increased expression of *CAT2 *mRNA, a critical arginine transporter, observed in our AD samples may further promote arginase activity. Interestingly, Hesse et al [12,54]. have shown that increased *AG1 *expression in schistosome egg-induced granulomas is associated with increased proline and polyamine production and promotes fibrosis in liver. Hesse et al [54] demonstrated that the re-induction of *NOS2 *expression or activity reduced the fibrotic load in the parasite-induced liver granulomatosis model. The upregulation of *AG1 *in AD, coupled with the loss of *NOS2 *mRNA, then, may have critical relevance to amyloid deposition in the extracellular matrix of the brain.

The *FIZZ1 *and *YM1 *genes also provide a link between alternatively activated macrophages and repair processes after infection or injury [10,70]. The protein product of *YM1 *induction is a novel mammalian lectin that binds saccharides and heparin/heparin sulfate on cell surfaces, but whose functions are largely unknown [70,75]. Hung et al [75] have suggested that *YM1 *helps to protect the extracellular matrix scaffold at sites of injury by reducing heparin sulfate degradation. *FIZZ1 *encodes a 9.4 kDa cysteine rich protein which was originally described in lung lavage fluids in a murine allergic pulmonary inflammation model [11]. Three *FIZZ *family members have been identified and are now known to be part of a new gene family of resistin-like molecules. As such, *FIZZ *proteins may contribute to insulin resistance during diabetes but they have also been linked to angiogenesis, to stimulation of collagen production and to inhibition of apoptosis [10,11,76]. Although a human homolog exists for *FIZZ1*, no direct human homologs have been identified for *YM1*. Our data, however, demonstrates that two closely related chitinase genes, namely *CHI3L1 *and *CHI3L2 *are overexpressed in AD brain. Both forms of chitinase 3-like proteins do not have enzymatic chitinase activity and instead, inhibit IL-1 and TNFα-mediated responses by blocking cell signaling [8].

In summary, immune cells within the brain display gene profiles that suggest heterogeneous, functional phenotypes that range from a pro-inflammatory, classical activation state to an alternative activation state involved in repair and extracellular matrix remodeling. These different functional phenotypes not only protect the tissue from invaders, but orchestrate and promote tissue reconstruction resulting in resolution of the injury. Repair processes mediated by alternative activation genes, however, can be associated with maintenance of disease and, in particular, enhanced fibrosis [6,12,77]. For example, diseases in the periphery that have fibrosis as a characteristic feature, such as *Schistosoma japonicum *egg induced fibrosis in the lung, *Schistomsoma mansoni *infection of the liver or idiopathic lung fibrosis show defective repair that, in fact, favors fibrosis [54,55,77]. Anti-inflammatory cytokine treatment under these circumstances worsens the fibrosis, while the re-establishment of NOS induction and activity reduces the fibrosis [12,77]. Neuroinflammation in AD is characterized by both degeneration and regeneration that occurs in a specific pattern of time and locale [53]. Studies on AD neuropathology implicate the presence of a defective repair process that is linked to the presence of Abeta peptides and amyloid fibrils [[53];78]. Our data presented here begin to build the case that alternative activated macrophages are present in AD brain and may contribute to a Th-2-linked, rather than a Th-1 linked, pathology. If true, then therapeutic approaches may need to consider this additional alteration of the immune response.

## Competing interests

The author(s) declare that they have no competing interests.

**Other Competing interests (not pertinent to the manuscript): **MPV is a principal in Cognosci, Inc.

## Authors' contributions

CAC designed the study, analyzed data, prepared figures and wrote the manuscript. RM performed Q RT-PCR experiments, analyzed data and contributed to the preparation of the manuscript; HS performed Q RT-PCR; QX prepared brain samples, performed Q RT-PCR and analyzed data; WVN provided transgenic mice and contributed to the preparation of the manuscript; MPV provided transgenic mice, participated in the study design and in the manuscript preparation.
